# Adrenal gland haemangioma, a rare entity difficult to differentiate from malignancy

**DOI:** 10.1093/bjrcr/uaae027

**Published:** 2024-08-08

**Authors:** Paola López Gómez, Miguel Paniagua Gonzalez, Angela García Pérez, Luis Alberto Mullor Delgado

**Affiliations:** Radiology Unit, Gregorio Marañón Hospital, Madrid 28007, Spain; Radiology Unit, Gregorio Marañón Hospital, Madrid 28007, Spain; Radiology Unit, Gregorio Marañón Hospital, Madrid 28007, Spain; Radiology Unit, Gregorio Marañón Hospital, Madrid 28007, Spain

**Keywords:** adrenal haemangioma, differential diagnosis, imaging, case report

## Abstract

Adrenal haemangioma is a rare benign vascular lesion, which is usually asymptomatic and it is typically discovered incidentally on radiographic imaging. Differential diagnosis from other benign or malignant adrenal neoplasms may be challenging, and in many cases, the diagnosis is only possible after surgical resection. We present a case of a 39-year-old female with abdominal pain in the upper right quadrant, who was referred to our hospital after incidentally discovering a mass above the right kidney on abdominal ultrasonography. MRI revealed an adrenal mass, with features not indicative of adenoma and suggestive of adrenal haemangioma, without ruling out other possible diagnoses such us phaeochromocytoma and adrenal cortical carcinoma. Biochemical tests did not reveal any endocrine dysfunction. The patient underwent adrenalectomy, and histopathological analysis confirmed a venous haemangioma. Adrenal gland haemangioma is an unusual vascular lesion, typically diagnosed incidentally during abdominal imaging. Certain radiologic features may raise suspicion for malignancy, making it difficult to distinguish them from a primary adrenal cortical carcinoma. They may also grow large, compressing surrounding structures and causing abdominal pain, or may rupture, leading to retroperitoneal haemorrhage. For these reasons, some authors recommend excision of all suspected adrenal haemangiomas, and in many cases, the final diagnosis is made only after surgical removal.

## Introduction

Haemangiomas are benign vascular lesions that most commonly affect the skin and the liver.^1^ Adrenal location is extremely rare and, mainly discovered incidentally on radiographic imaging. It represents 0.01% of adrenal tumours, with nearly 70 surgical cases reported in different databases since 1955.^2^ The clinical significance of adrenal haemangiomas lies in their difficulty to differentiate from adrenal cortical cancer, as both can present similar clinical and imaging features. Thus, preoperative diagnosis of adrenal haemangioma remains challenging, with the final diagnosis often established after surgical resection and pathological examination.^3^

We present a case of an adrenal haemangioma incidentally diagnosed following abdominal ultrasound in a 39-year-old female with upper right quadrant abdominal pain. We discuss the diagnosis work-up, treatment, final findings, and patient follow-up.

## Case presentation

**Figure 1.**
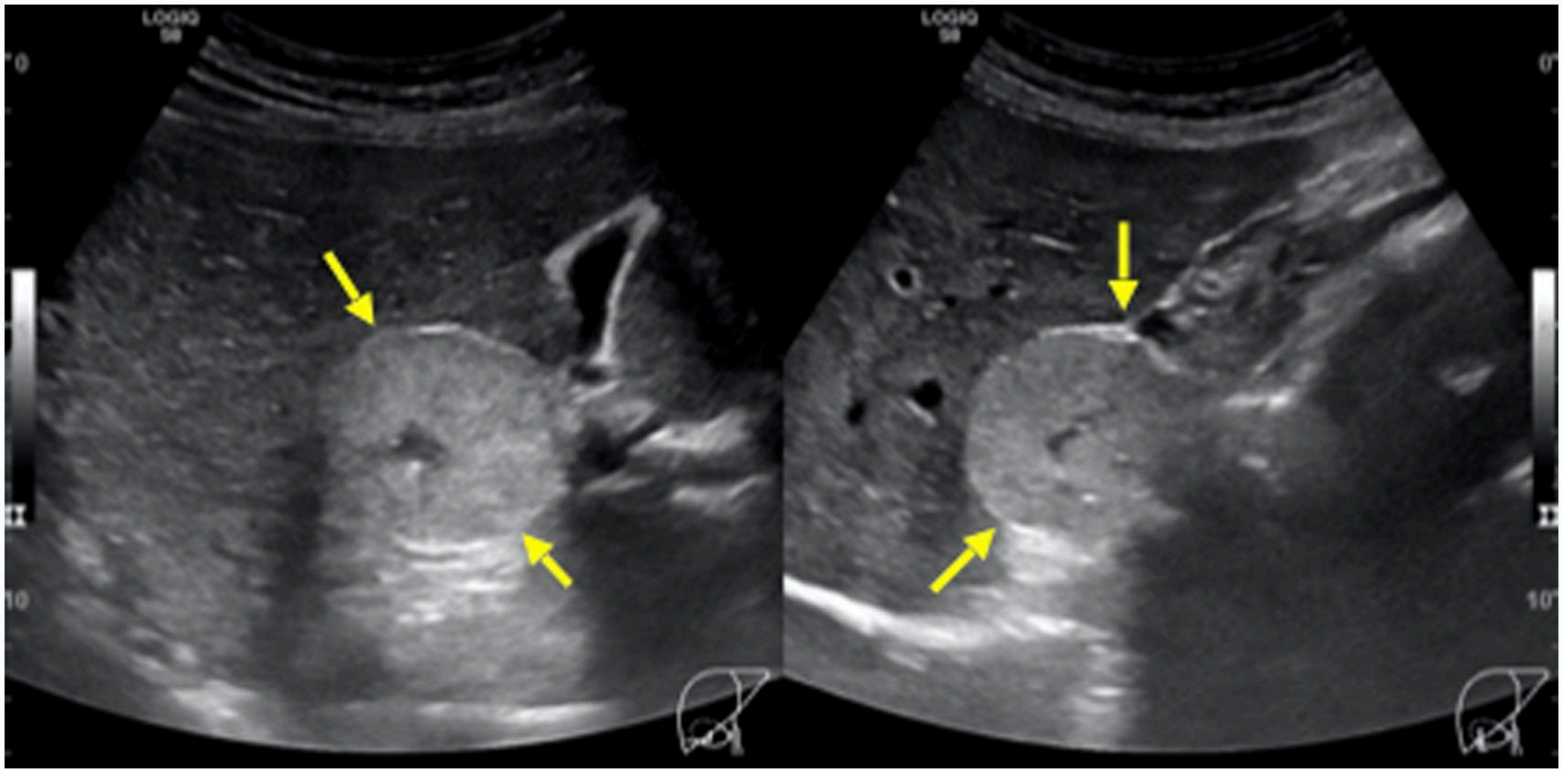
Abdominal ultrasound in axial and sagittal planes at the right upper quadrant reveals a hyperechoic mass with clearly defined margins (yellow arrows), adjacent to the inferior right hepatic lobe.

A 39-year-old female, with a medical history or rheumatoid arthritis, oesophagitis, and gastritis, was referred to our hospital following an incidental discovery of a solid mass above the right kidney during abdominal ultrasonography (Figure 1). This study was prompted by the patient’s complaint of abdominal pain in the right upper quadrant to her general practitioner. The radiologist recommended further investigation with an abdominal MRI.

**Figure 2.**
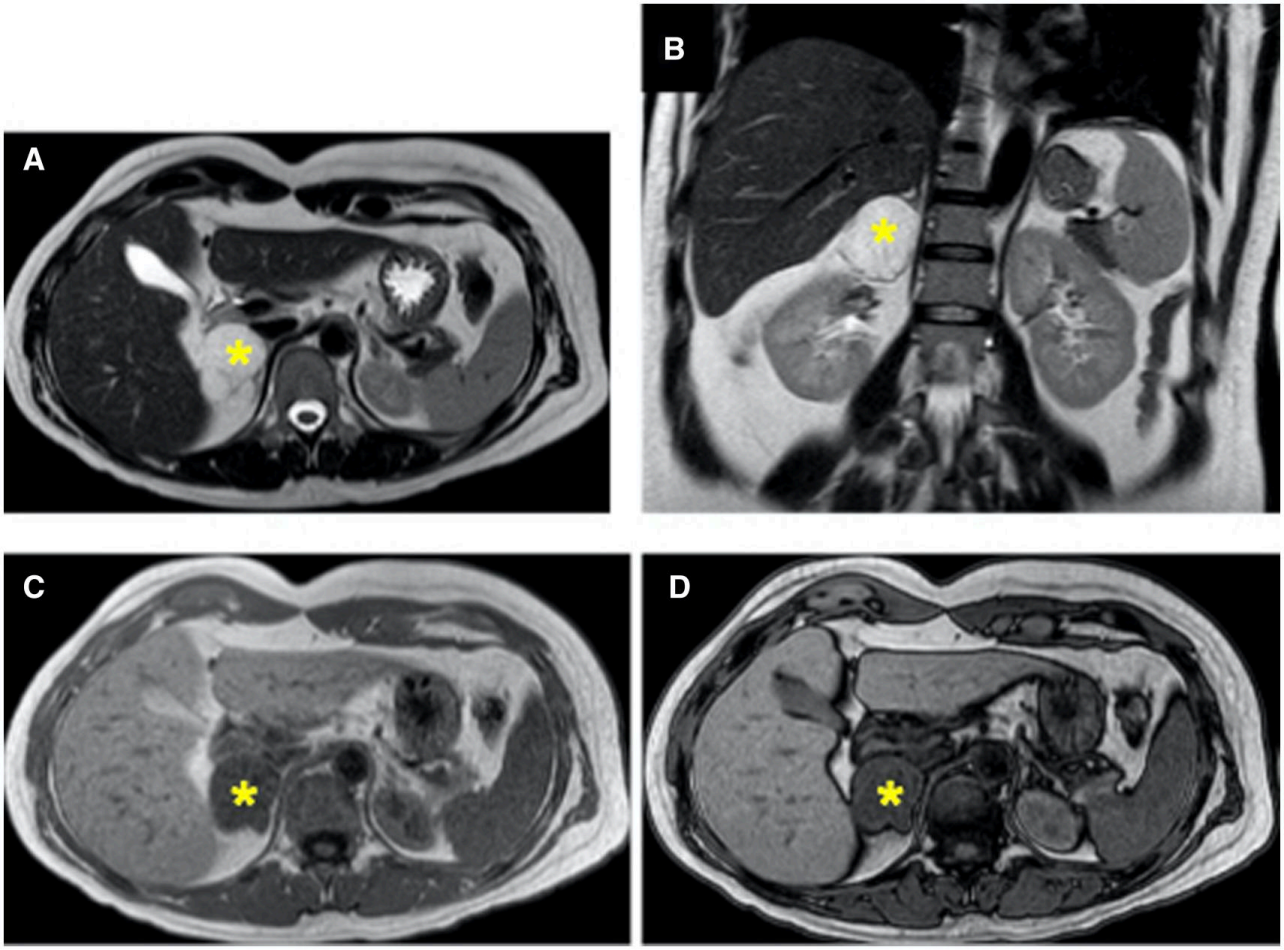
Abdominal MRI shows a right adrenal mass (yellow asterisk) with high signal intensity on T2-weighted axial (A) and coronal (B) images, and an absence of signal intensity drop out on out-of-phase compared with in-phase images (C and D).

**Figure 3.**
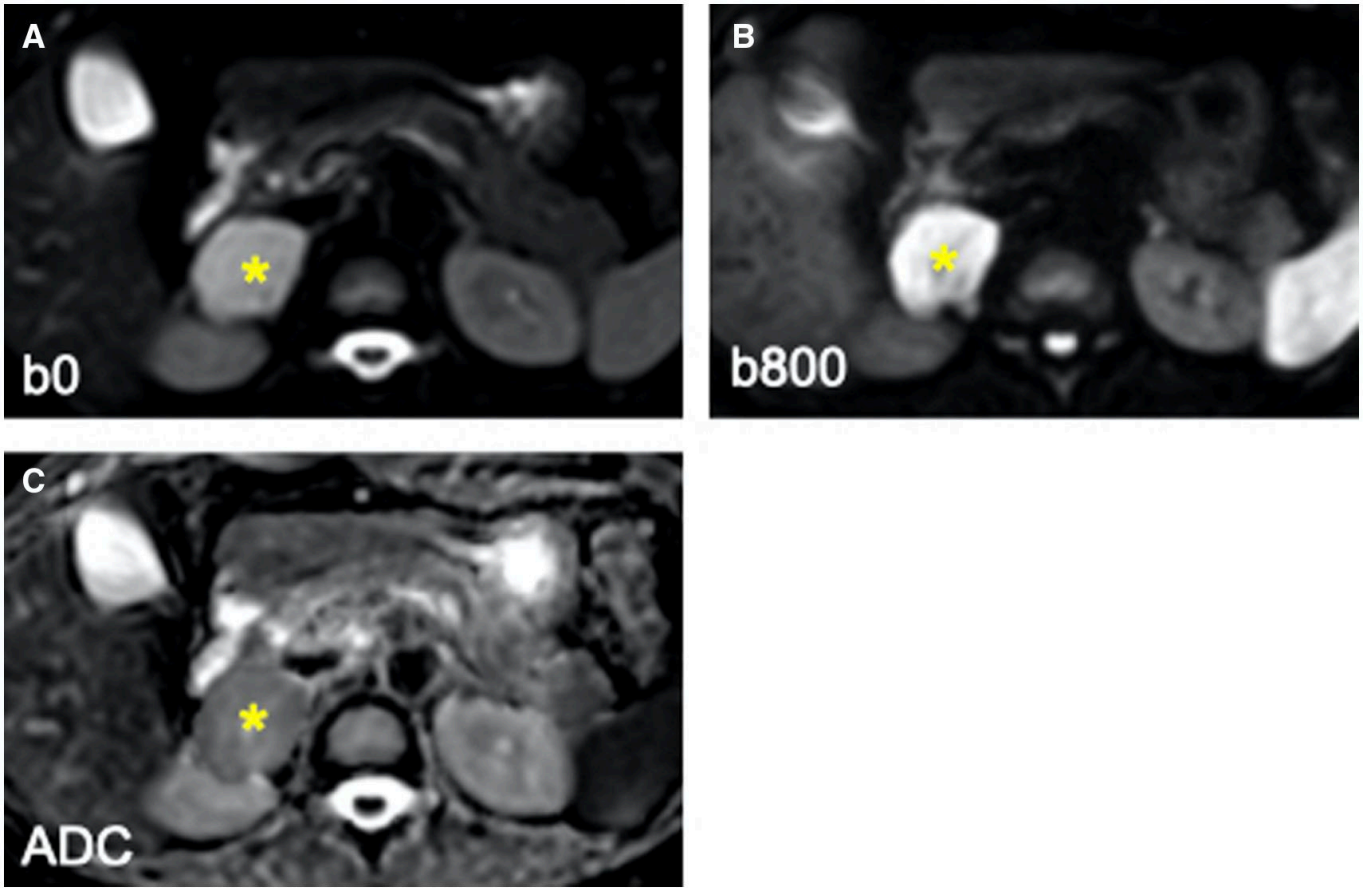
Diffusion-weighted imaging with b-values of 0 (A) and 800 (B), and apparent diffusion coefficient (ADC) (C) reveals no significant diffusion restriction of the lesion.

**Figure 4.**
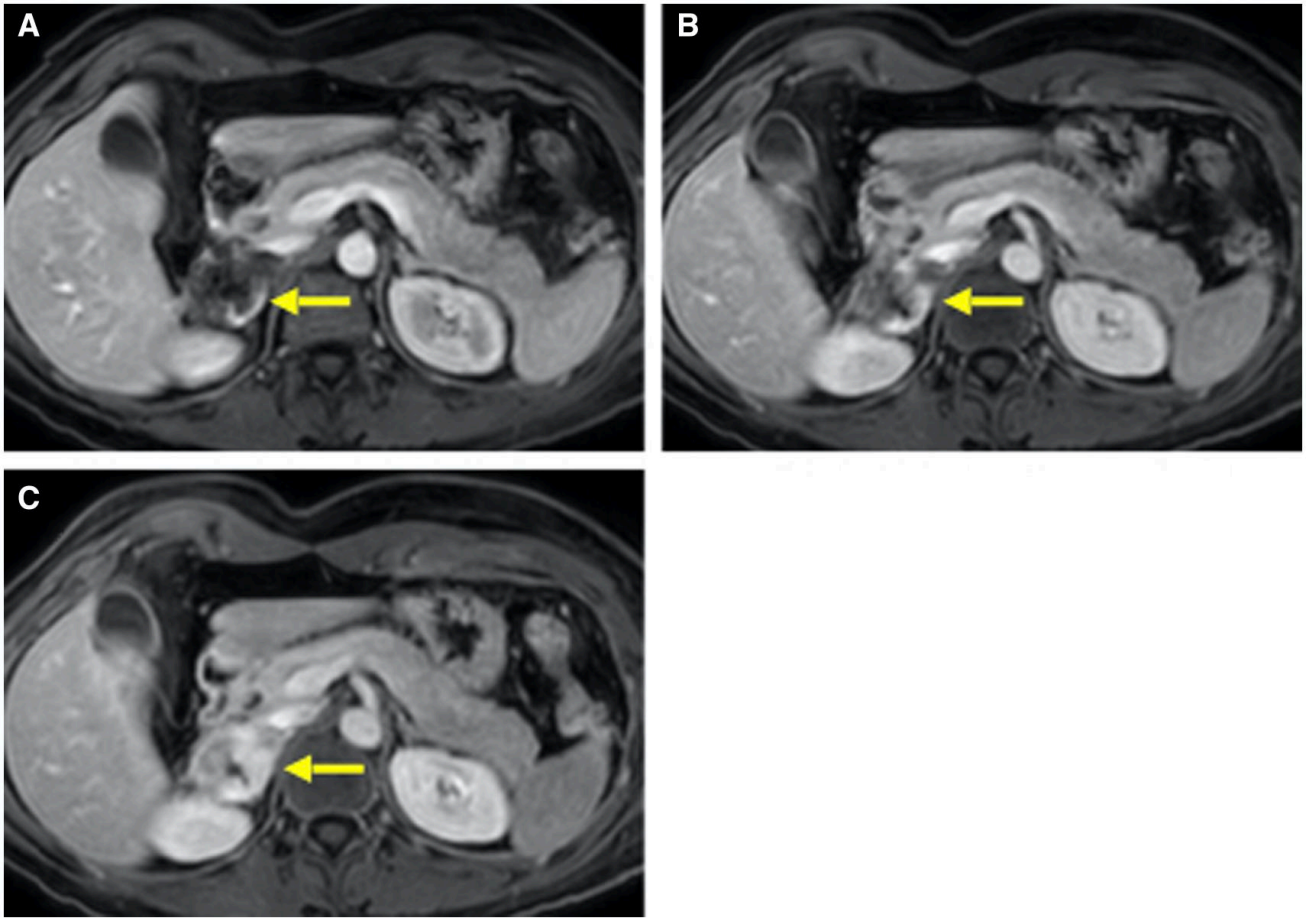
The lesion exhibit peripheral discontinuous enhancement in arterial phase (A) with gradual centripetal filling in venous (B) and late phases (C).

Subsequently, abdominal MRI was conducted a month later, revealing a 4.5 cm solid mass in the right adrenal gland. The mass displayed hypointensity on T1-weighted images, hyperintensity on T2-weighted images, absence of signal intensity drop-out in out-of-phase compared with in-phase images, and moderate restriction of intralesional molecular water diffusion in diffusion-weighted imaging (Figures 2 and 3). Peripheral enhancement in the arterial phase with gradual centripetal filling in venous and late phases was noted (Figure 4). These radiological features were not consistent with adenoma and were suggestive of adrenal haemangioma, with pheochromocytoma and adrenal cortical carcinoma included in the differential diagnosis.

Clinical examination was unremarkable, and the patient did not exhibit any clinical manifestations of adrenal medullary or cortical hyperfunction. Laboratory tests, including adrenal levels, plasma aldosterone, and metanephrines, were within normal limits (Adrenocorticotropic hormone (ACTH) 21.8 ng/L, cortisol 11 g/dL, aldosterone 15.9 ng/dL, normetanephrine 1023 nmol/L, metanephrine 710 nmol/l, methoxytyramine 649 nmol/L).

Upon reviewing all diagnostic findings and considering the size of the mass and the non-specific radiological features, in accordance with our hospital protocol aligned with international guidelines,^4^ a decision was made to proceed with surgical resection.

Five months later, the patient underwent laparoscopic right adrenalectomy. Intraoperatively, the mass was located at the inferior margin of the adrenal gland, with a diameter of approximately 3 cm and no evidence of local infiltration. The procedure was uncomplicated and the postoperative course was uneventful, with the patient being discharged home on postoperative day 2.

The pathological examination revealed a 4-cm blackish congestive mass emerging from the right adrenal gland. Histologically, it was composed of multiple capillary vascular structures forming lobules, with thin endothelial borders and no atypia or other signs of malignancy.

At 12 months follow-up, the patient remains well with no evidence of recurrence.

## Discussion

Adrenal haemangiomas are rare vascular malformations typically discovered incidentally on abdominal imaging.

According to the latest classification from the International Society for the Study of Vascular Anomalies (ISSVA), vascular malformations are categorized into capillary, lymphatic, venous and arteriovenous malformations, with cavernous haemangiomas being the most prevalent type in the adrenal gland.^5^

Main age at presentation is between the ages of 50 and 70 years, being females more frequently affected than males (female:male ratio 2:1).^6^

They typically exhibit unilateral growth and measure less than 2 cm in size. These lesions are usually asymptomatic and non-functioning, although a few cases of secreting adrenal haemangiomas have been documented. However, adrenal haemangiomas can occasionally grow large, causing abdominal pain due to compression of surrounding structures, or rupture leading to retroperitoneal haemorrhage.

Radiologically, adrenal haemangiomas exhibit well-delimitated soft tissue attenuation masses on CT scans. With contrast administration, they enhance similarly to haemangiomas elsewhere, heterogeneously and mainly peripheral, with gradual centripetal filling. MRI is often helpful, demonstrating lesions that are hypointense relative to the liver on T1-weighted images, hyperintense in T2 sequences, with peripheral enhancement and gradual filling after gadolinium administration.

However, diagnosis can be challenging in the absence of centripetal enhancement, as this feature may also be observed in other adrenal lesions.^1^

Furthermore, distinguishing haemangiomas from a malignant mass can be difficult due to overlapping imaging features such as large size, lack of microscopic fat, delayed contrast washout, heterogeneity, calcification, and necrosis.

Biochemical test usually do not indicate any endocrine dysfunction. Differential diagnosis should consider other adrenal masses, especially adenomas, the most common ones. They can be detected on a nonenhanced CT by showing attenuating values less or equal to 10 HU (lipid-rich adenomas), or using washout CT or MRI techniques, as they typically enhance rapidly and have rapid washout.

Malignant adrenal lesions should always be considered, and they enhance rapidly with prolonged washout. Metastasis may lack specific imaging characteristics, although the primary tumour is usually known previously. Adrenal carcinomas tend to be heterogeneous and large, and half of them show signs of cortical hyperfunction.

Phaeochromocytomas can be confused with both benign and malignant lesions, as they exhibit a variety of enhancement patterns. Most of them present washout characteristics similar to those of malignant adrenal lesions, but they can also meet the same washout criteria as cortical adenomas.^7^^,^^8^ On MRI, most of them exhibit high signal intensity on T2-weighted images (light bulb sign).^9^ They usually manifest clinically due to catecholamine hypersecretion, and iodine 123 metaiodobenzylguanidine whole body scintiscan single-photon emission-computed tomography could help rule out their presence.^5^

Macroscopically, adrenal haemangiomas appear as brownish soft masses, with microscopic evaluation revealing multiple capillary vascular structures of variable sizes and shapes, with a regular endothelial border and separated by thick fibrous septa of variable thickness. Calcifications and thrombi may also be present.

Due to the inability to definitively rule out malignancy and the potential for complications, surgical excision is often recommended for suspected adrenal hemangiomas,^10^ with the final diagnosis confirmed by pathological examination after surgical removal.

## Conclusion

Adrenal haemangioma, though rare, should be considered in the differential diagnosis of nonfunctioning adrenal masses, alongside adenomas, pheochromocytomas, adrenal cortical cancer, and metastasis.

Radiologic features can sometimes mimic malignancy, necessitating surgical resection for definitive diagnosis and management of potential complications.

## Learning points

- Adrenal haemangioma is a benign vascular lesion usually asymptomatic but can present potential life-threatening complications like retroperitoneal bleeding.
- It should be considered in the list of possible causes of an adrenal mass, as its radiological appearance is often challenging to distinguish from a malignant condition.
- Surgical removal is generally required to prevent complications and to exclude malignancy.
